# Development of a Novel Algorithm for Tip Fold-Over Detection in Cochlear Implants and Evaluation on Bench and Multiple Clinical Data Bases

**DOI:** 10.3390/audiolres15050118

**Published:** 2025-09-18

**Authors:** Mehrangiz Ashiri, Tony Spahr, Azret Botash, Ashish Mehta, Jordan J. Varghese, Craig A. Buchman, Andrea J. DeFreese, Patrick Boyle, Matthew Miller, Syed F. Ahsan, Christopher Danner, Kyle P. Allen, Loren Bartels, Kanthaiah Koka

**Affiliations:** 1 Research and Technology, Advanced Bionics LLC, Valencia, CA 91355, USA; 2 Department of Otolaryngology, Washington University, St. Louis, MO 63130, USA; 3 Department of Hearing and Speech Sciences, Vanderbilt University Medical Center, Nashville, TN 37232, USA; 4 Otolaryngology-Head and Neck Surgery, Sanford Health, Fargo, ND 58104, USA; 5 Head & Neck Surgery Department, Southern California Kaiser Permanente Medical Group, Anaheim, CA 92806, USA; 6 Tampa Bay Hearing & Balance Center, Tampa, FL 33606, USA

**Keywords:** tip fold-over, electric field imaging (EFI), cochlear implants, diagnostic algorithm, Advanced Bionics, pre-curved, lateral wall, electrode array

## Abstract

**Objectives:** Tip fold-over (TFO) is a rare but critical occurrence in cochlear implant procedures where the electrode array folds back on itself within the cochlea, compromising programming and device performance. Timely intraoperative detection is essential for immediate correction and optimal placement. Electric field imaging (EFI) has shown promise for identifying TFO both intra- and post-operatively. This study evaluates the performance of a TFO detection algorithm implemented in Target CI (Version 1.6) using Advanced Bionics’ cochlear implant systems, validated through bench and patient datasets. **Methods:** Sample data included (1) bench testing with a plastic cochlea and human temporal bones with and without induced TFOs, confirmed visually or radiographically; (2) intraoperative EFI measurements recorded using the AIM™ system, with electrode placement confirmed through imaging; and (3) historical EFI recordings from the Target CI DataLake, which lacks imaging and programming metadata. The TFO algorithm’s performance was evaluated by assessing its sensitivity and specificity using these datasets. **Results:** The TFO algorithm achieved 100% sensitivity and specificity in bench models and intraoperative EFI with imaging-confirmed placements. Among 226 intra-op cases, four TFOs were confirmed by imaging, and all were correctly identified by the algorithm. In the large set of DataLake cases (14,734 implants), 0.80% were flagged as potential TFOs. TFO prevalence was higher with pre-curved arrays (1.22%) than straight lateral wall arrays (0.32%). **Conclusions:** The TFO algorithm showed high reliability with 100% sensitivity and specificity using routine clinical EFI data. While not a replacement for imaging, the TFO algorithm serves as a fast, accessible tool to alert clinicians to potential TFOs.

## 1. Introduction

Cochlear implant electrode tip fold-over (TFO) is a rare but clinically significant complication that compromises implant effectiveness and often requires surgical revision. TFO occurs when the electrode array folds back on itself during insertion, disrupting the electrode-to-cochlea interface and leading to several adverse outcomes. These include reduced insertion depth, abnormal spread of excitation, unintended co-stimulation of the facial nerve, additional cochlear trauma, and consequently hearing restoration challenges. The occurrence of TFO is influenced by factors such as the insertion technique, type of electrode array, and patient-specific anatomical characteristics, including cochlear duct size, shape, and the presence of any ossification. Notably, TFO is more commonly observed with pre-curved electrode arrays compared to straight lateral wall designs [1,2,3]. Using different techniques to verify the presence of TFO, previous studies have reported TFO occurrence rates ranging from 1.67% to 12% in pre-curved electrode arrays and from 0.23% to 14.29% in lateral wall electrodes [1,3,4,5,6,7,8,9,10,11,12,13,14]. The rate of TFO varied based on study population size and the method used to verify electrode placement. Notably, the pre-curved electrode has been more frequently associated with TFO [2,10,11,13] than straight lateral wall electrode.

The identification of electrode tip fold-over (TFO) in cochlear implants typically relies on a combination of intraoperative and postoperative assessments, incorporating both objective and subjective methods. Objective techniques include imaging modalities such as X-ray, computed tomography (CT), and fluoroscopy, which are generally more accurate in detecting TFO. However, these methods present notable limitations—they are time-consuming, expensive, and involve exposure to ionizing radiation. Moreover, electrode array artifacts can obscure the clarity of the images, making accurate diagnosis more challenging. Intraoperative imaging, while potentially useful, is not routinely performed in many surgical centers due to these constraints, thereby limiting the ability to correct TFO during the initial implantation.

Electrophysiological measures such as electrically evoked compound action potentials (eCAPs) offer an alternative means of detecting TFO. These recordings assess neural responses to electrical stimulation at individual electrode contacts. In cases of TFO, the spatial spread of excitation is often disrupted, resulting in broadened or asymmetrical activation patterns near the fold. This occurs because the folded portion of the array stimulates non-target cochlear regions or unintended neural populations, producing atypical current distribution.

Subjective assessments, such as pitch ranking tasks, can also reveal signs of TFO. Patients may report irregularities in pitch perception along the electrode array, particularly near the site of the fold. For example, apical electrodes might produce higher-pitched percepts than more basal ones, indicating a reversal or distortion in the tonotopic organization. This pitch anomaly serves as a qualitative indicator of TFO. However, behavioral techniques are not suitable for intraoperative use and are generally not feasible in infants or very young children.

Critically, the optimal window for detecting TFO is intraoperatively—during the initial cochlear implantation procedure—when the electrode array can still be repositioned or replaced without necessitating a second surgery. Yet, given the low incidence of TFO and the high cost and complexity of imaging in the operating room, routine imaging after every insertion is impractical. While eCAP and pitch-ranking methods are helpful adjuncts, they suffer from limitations in sensitivity and are dependent on the integrity of the auditory nerve, reducing their utility in certain populations.

Taken together, these limitations underscore the need for a rapid, accurate, and safe intraoperative diagnostic method for detecting TFO—one that is independent of cochlear health and feasible across patient populations, including young children.

Electric field imaging (EFI), also referred to as Trans Impedance Matrix (TIM) or Impedance Field Telemetry (IFT) by various cochlear implant manufacturers, has emerged as a promising tool for evaluating electrode placement. EFI is obtained by delivering a known electrical current to a specific electrode contact in the implant’s array while recording the amount of voltage appearing on both stimulating and non-stimulating electrode contacts through back telemetry. These voltages are used to generate an impedance matrix that provides insights into the electrical properties and spatial arrangement of the electrode array within the cochlea [15,16,17].

Using the impedance matrix as the reference point, Vanpoucke et al. proposed a method to estimate electrode array placement within the cochlea by deriving relative electrode positions in a 2D space [15]. The estimated electrode contacts’ positions can identify misplacements in the cochlea. Building on this approach, we developed an algorithm specifically designed to identify the occurrence of a TFO using EFI measurements obtained through two regular clinical tools: Target CI programming software (Version 1.5) and the AIM™ (Version 1.1) system (Advanced Bionics LLC, Valencia, CA, USA). This study aimed to evaluate the reliability of the proposed TFO algorithm in both experimental and real-world scenarios, assessing its accuracy in detecting TFO instances using data from bench tests and clinical cases, with and without imaging.

## 2. Methods

Advanced Bionics offers a variety of electrode array designs, including HiFocus Helix, HiFocus 1J, HiFocus Mid Scala, and HiFocus SlimJ, to accommodate diverse cochlear anatomies and surgical preferences. The HiFocus Helix and HiFocus Mid-Scala are pre-curved electrode arrays, whereas HiFocus 1J and HiFocus SlimJ are straight electrode arrays. Despite the variations in design, all electrode array types feature 16 individual electrode contacts.

### 2.1. Description of EFI

**Figure 1:**
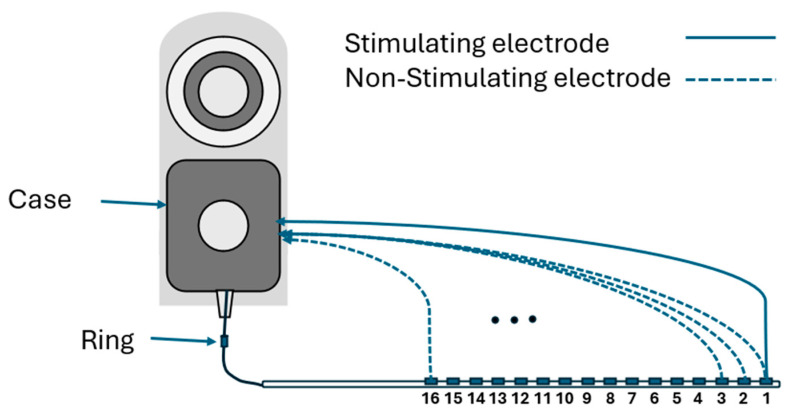
Impedance measurements obtained using a cochlear implant in a monopolar configuration. Electrode 1 is stimulated, and voltages are recorded across electrodes 1 to 16. This procedure is repeated sequentially for electrodes 2 through 16, resulting in a 16 × 16 voltage matrix. The voltage values are subsequently divided by the applied current to derive the impedance matrix.

**Figure 2:**
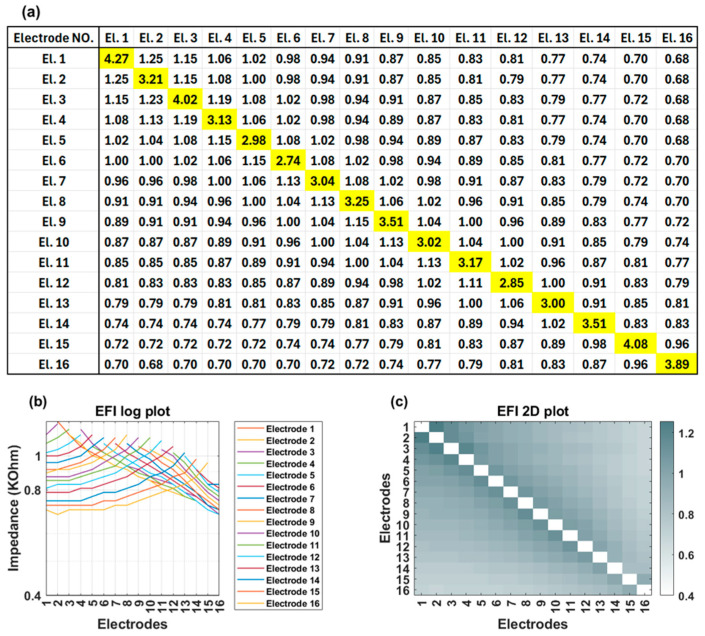
(**a**) EFI matrix of a cochlear implant user with normal impedance levels and no TFO. The diagonal elements represent the impedances measured from the stimulating electrodes (main impedances), while the off-diagonal elements correspond to the impedances recorded from non-stimulating electrodes (cross-impedances). All impedances are in kΩ unit; (**b**) The EFI log plot illustrates the cross-impedances for each row, where under normal conditions, the cross-impedances decrease with increasing distance from the stimulating electrode. (**c**) The heat map (2D plot) visually represents this gradual decline as the distance from the stimulating electrode increases.

EFI measurements are obtained using regular clinical tools (Target CI and the AIM™ system). For Advanced Bionics, EFIs are measured in the background when a clinician measures regular electrode contacts impedances. To produce the EFI matrix, a known electrical current pulse (amplitude of 32 µA, pulse width 17.96 µs per phase, biphasic, cathodic-leading) is delivered to a specific electrode contact in the cochlear implant array, and the resulting voltages appearing on each of the 16 electrode contacts within the cochlea are recorded. Note this involves recording from stimulating and non-stimulating electrode contacts. Stimulation and recording use a monopolar circuit configuration where current is introduced into the cochlea and returned via the remote ground electrode, and voltage appearing on each intra-cochlear electrode contact is recorded with respect to the ground electrode. The ground contact may be either the case or ring electrode [18] (Figure 1). This process is repeated systematically for each electrode contact in turn to generate a detailed matrix. The measured voltage values are converted into an impedance matrix by dividing them by the applied current. For an electrode array with 16 contacts, the resulting impedance matrix has a dimension of 16 × 16, comprising 256 elements. This matrix offers valuable insights into the electrical properties of the cochlea and spatial arrangement of the electrode array within the cochlea. The leading diagonal elements of the impedance matrix represent the monopolar electrode contact impedances of the stimulating electrodes, while the off-diagonal elements correspond to the impedances of the non-stimulating electrodes (Figure 2a). The leading diagonal impedance values are displayed by Target CI and the AIM™ system and are used for clinical fittings, while off-diagonal values are not usually displayed. Figure 2b,c offer visual representations of EFI data through a line (log) plot and a heat (2D) map, respectively. Notably, for a normally positioned electrode array, the off-diagonal impedance values corresponding to non-stimulating electrodes exhibit a monotonic decrease with increasing distance from the stimulating electrode contact.

**Figure 3:**
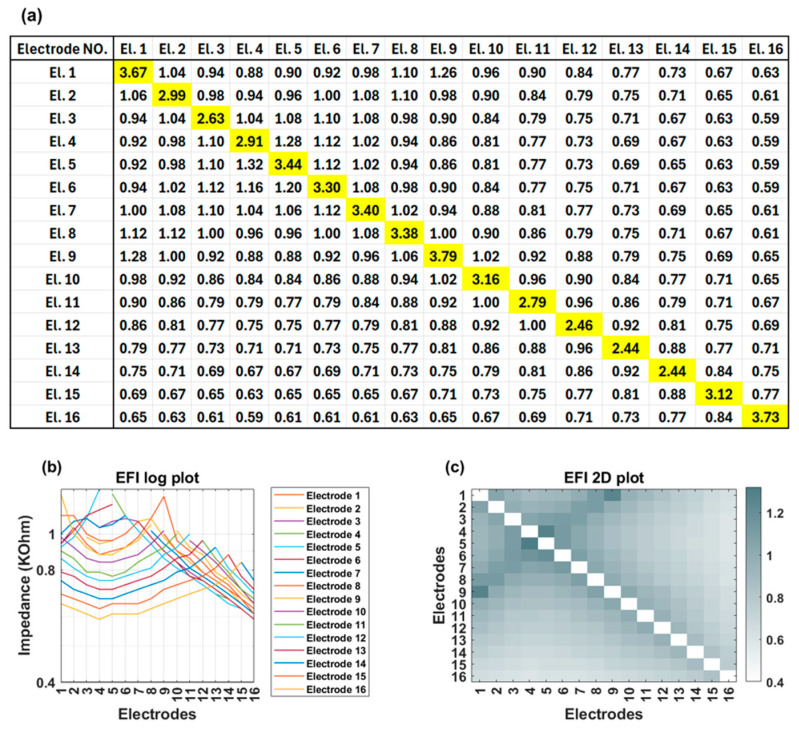
(**a**) EFI matrix of a cochlear implant user with fold-over on electrode 5; (**b**) EFI log (line) plot shows a distinct symmetry on either side of the fold-over location (electrode 5); (**c**) The heat map (2D plot) reveals an increase in cross-impedance values following an initial decrease as the distance from the stimulating electrode increases.

Figure 3 shows an example of EFI measured for an implant recipient who has a TFO on her electrode array. In the presence of a tip fold-over, the cross-impedance values exhibit an initial decrease, followed by an increase, and then a subsequent decrease, depending on the location of the fold-over (Figure 3a). This is visually identifiable in the line graph through a distinct symmetry observed on either side of the fold-over location (electrode contact 5 in Figure 3b). A TFO is observed as a cross-diagonal pattern (running from bottom left to top right) at the apical section in the 2D EFI graph (Figure 3c).

### 2.2. Description of TFO Algorithm

The impedance matrix described above was used as input to the TFO algorithm. The first step in the TFO algorithm is the pre-processing of the EFI matrix to identify and handle any open, shorted, or invalid electrode contacts. The pre-processing procedure includes the following steps:

#### 2.2.1. Initial Impedance Check

Main impedance values in the 16 × 16 matrix are evaluated. If any impedance value exceeds 30 kΩ (indicative of an open electrode), falls below 0.5 kΩ (indicative of a short to ground), or if there is a partial short between a pair of electrode contacts, the associated row and column are removed from the matrix.

#### 2.2.2. Validation of Data Integrity:

The algorithm is not executed if any of the following conditions are met:

- More than three electrode contacts exhibit out-of-range values.
- Any cross-impedance in the first row exceeds 5 kΩ, which may indicate insufficient contact between the implant’s case ground and surrounding tissue. This condition can be temporarily observed in the operating room when an air pocket prevents the implant’s case ground from making direct contact with the surrounding tissue.

These pre-processing steps are critical to ensure reliable and effective application of the algorithm.

Using principal component analysis (PCA), the first two components (PC1 and PC2) are extracted, which capture the majority of the variance in the original data. The location of any TFO is determined based on the angular distance between the index of the minimum value in the principal component and the rest of the electrode contact indices.

**Figure 4:**
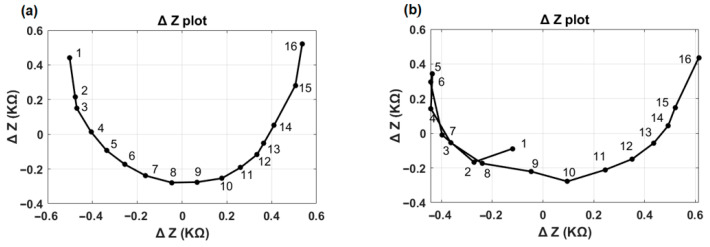
∆Z plot in which PC1 and PC2 have been plotted against each other. (**a**) Normal case with a smooth, continuous curvature; (**b**) TFO case with sharp bend on electrode 5.

Figure 4 illustrates the first two principal components (ΔZ plot) for the normal and TFO examples shown in Figure 2 and Figure 3. In the normal case (Figure 4a), the ΔZ plot exhibits a smooth, continuous curvature without abrupt deviations. In contrast, the TFO case (Figure 4b) reveals a distinct inflection point or sharp bend at electrode 5, highlighting the presence of a tip fold-over’s characteristics. This sharp bend is associated with the reversal of the normal impedance value decay along the rows of the EFI matrix, or minimum angle of the vector appearing at an electrode contact different from electrode contact 1. The TFO algorithm identifies both the presence of a TFO and the location of the TFO.

### 2.3. Evaluation of the TFO Algorithm

Two distinct bench experiments were conducted to evaluate the performance of the TFO detection algorithm: (1) a plastic cochlea submerged in a saline bath and (2) a human temporal bone experiment. Additionally, to further evaluate the algorithm’s performance, patient data with and without imaging were also analyzed.

#### 2.3.1. Plastic Cochlea

Bench tests were conducted using a plastic cochlea that is representative of a human cochlea. The plastic cochlea was manufactured using a 3D printer (Stratasys Direct Manufacturing, Valencia, CA, USA-91355) based on multiple representative human cochleae microCT images. This plastic cochlea has a scala size representative of human cochleae. The implant types used for bench testing were the HiRes Ultra 3D implant body with HiFocus Mid Scala or SlimJ electrode arrays, and the HR90K Advantage implant body with a HiFocus 1J electrode array. EFI measurements were obtained using Target CI under two conditions: (1) a normal full electrode insertion and (2) a fold-over in the array, occurring between electrode contacts 3 and 7. Twenty-three EFI measurements were collected with different conditions to capture different variabilities of fold-over at different electrode locations. These EFI measurements were subsequently input into the TFO algorithm to identify the presence of TFO and compared with actual visual inspections for sensitivity and specificity.

#### 2.3.2. Temporal Bone

Bench tests were also performed using half cadaveric heads (temporal bones) to mimic real surgical measurements. These specimens were freshly frozen and thawed immediately before the experiments. The cadaveric specimens were drilled in a manner representing a typical CI surgery and their cochleae were opened to allow insertion of an electrode array via the round window. They were then utilized to assess the behavior of cochlear implant electrode arrays under controlled insertion conditions. Two HiRes Ultra 3D devices, one with a HiFocus Mid-Scala electrode array and one with a SlimJ electrode array, were used for testing. The study included both normal electrode insertions and deliberately created tip fold-over scenarios. Electrode positioning was verified using X-ray imaging, and EFI measurements were conducted for each condition using the Target CI clinical software. Each electrode array was inserted with a normal and a forced tip fold-over in each temporal bone. A total of eight recordings were performed, comprising two normal insertions and two tip fold-over insertions for each temporal bone. The TFO algorithm identified the presence or absence of TFO. The TFO algorithm’s categorization was compared with the corresponding X-ray image for each insertion to verify the sensitivity and specificity of the algorithm.

#### 2.3.3. Clinical Evaluation 1—EFI with Imaging Available

EFI data from either the AIM™ system or Target CI (Version 1.5), along with imaging, either CT or plain film X-ray, were available for 226 implant recipients. Of these 226, 115 received the HiFocus Mid-Scala electrode array, while 111 received the SlimJ electrode array. The implanting surgeon reviewed the CT scans and documented the presence of any TFO, which was then compared with the results generated by the TFO algorithm. These data were obtained either during standard intraoperative care or, in select cases, through device integrity assessments performed by Advanced Bionics clinical staff when imaging at a center revealed evidence of a TFO.

#### 2.3.4. Clinical Evaluation 2—EFI Without Imaging

To further assess the effectiveness of the TFO detection algorithm, we analyzed a large database of EFI data. This data is derived from real patients rather than bench testing; however, due to the size of the database and variability in standard of care across clinics, imaging was not available to confirm electrode placement. The goal was to compare the rate of TFOs found by the TFO algorithm against the percentage reported in prior literature. The EFI data were collected between December 2023 (after Target CI version 1.5 release) and December 2024 and were extracted from Target CI DataLake, a comprehensive platform that stores both structured and unstructured data. The data from DataLake include implant and electrode types and impedance (EFI) measurements which can be retrieved in customizable formats using data mining algorithms. This database lacks records on fitting history and cochlear anatomy. The implantation dates ranged from May 2000 to December 2024, encompassing all electrode types introduced over the past 24 years. A total of 52,276 EFI recordings were retrieved. The data were sorted chronologically, retaining only the most recent recordings, resulting in a dataset of 14,734 unique implant cases for evaluation. The TFO detection algorithm was then applied to this dataset to evaluate TFO occurrence. These data were also used to compare TFO occurrence rate between pre-curved (HiFocus Mid Scala and HiFocus Helix) and straight lateral wall electrode arrays (1J and SlimJ).

## 3. Results

### 3.1. Controlled Bench Testing

#### 3.1.1. Plastic Cochlea

**Figure 5:**
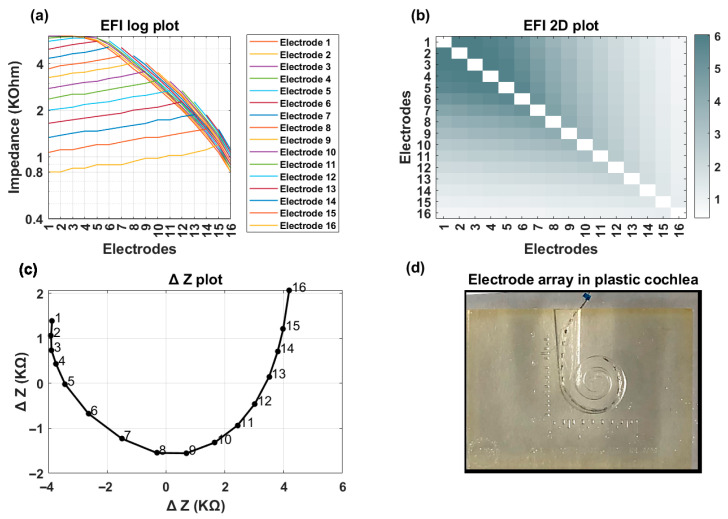
Normal electrode insertion simulated with plastic cochlea. (**a**) line plot; (**b**) heat map; (**c**) ∆Z plot; and (**d**) placement of electrode array inside plastic cochlea.

**Figure 6:**
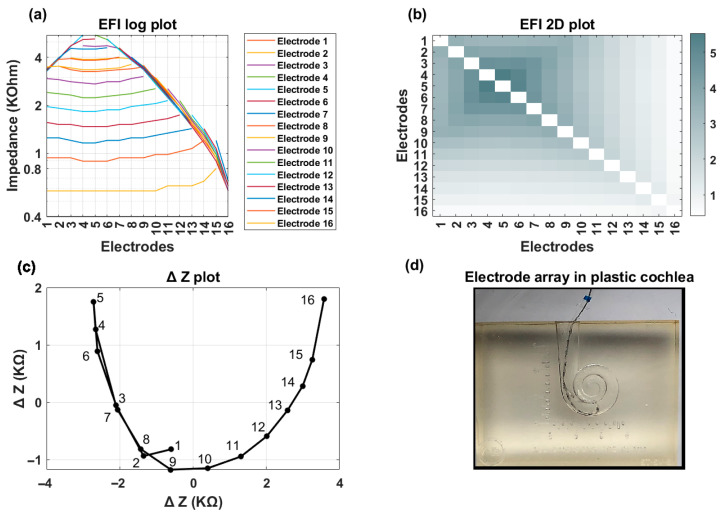
TFO condition simulated with plastic cochlea. (**a**) line plot; (**b**) heat map; (**c**) ∆Z plot with a sharp bend on electrode 5; and (**d**) placement of electrode array inside plastic cochlea.

Figure 5 and Figure 6 illustrate examples of normal and TFO scenarios tested using plastic cochlea. Note that higher cross-impedance values are observed in plastic cochlea bench settings compared to patient data, which typically range between 0.5 and 2 kΩ. However, this difference in value does not influence the overall pattern. The line and heat map plots (Figure 5a,b) demonstrate a gradual decrease in cross-impedance values as the distance from the stimulating electrode increases. Furthermore, the ∆Z plot (Figure 5c) exhibits a smooth curvature without any sharp bends. Figure 5d shows the lateral wall location of the electrode array within the plastic cochlea.

Figure 6 shows an example of TFO in plastic cochlea. In the presence of a TFO, the line and heat map plots fail to exhibit a monotonic decrease, with noticeable deviations depending on the location of the fold-over (Figure 6a). Additionally, the ΔZ plot demonstrates a pronounced inflection/curvature at electrode 5 (Figure 6b). Figure 6c illustrates the electrode array’s fold-over within the plastic cochlea.

#### 3.1.2. Temporal Bone

Table 1 presents the algorithm’s performance in terms of sensitivity and specificity. As shown, the algorithm accurately classified the EFI data for all eight temporal bone insertions: four with and four without a TFO.

### 3.2. Clinical Evaluation

#### 3.2.1. EFI with Imaging Available

Among the 226 cases evaluated, TFO was confirmed in four instances (1.77%). Of these, all four involved the pre-formed Mid Scala electrode array type, meaning that no TFO was associated with the lateral wall SlimJ electrode array type. Two TFOs were identified from intra-op measurement and were immediately corrected. The other two TFOs were identified from post-op EFI data and imaging and corrected by reimplantation (imaging or EFI data were not collected intraoperatively for the latter two cases, as they were not part of the standard of care at the two respective clinics). Table 2 displays the sensitivity and specificity of the TFO algorithm applied to the clinical dataset of 226 cases. High reliability with 100% sensitivity and 100% specificity was also observed with intra-op measured EFI with respect imaging data.

**Figure 7:**
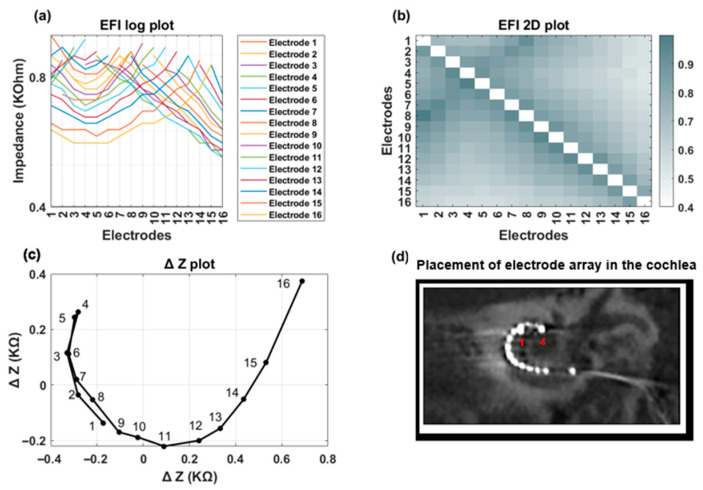
A clinical case with confirmed TFO on electrode 4. Measurements were taken post-op. (**a**) The line plot reveals a distinct symmetry on either side of the fold-over location (electrode 4); (**b**) The heat map highlights crosstalk between apical and less-apical electrode contacts; (**c**) The ∆Z plot demonstrates a sharp bend on electrode 4; (**d**) The CT image confirms the precise location of the TFO on electrode 4.

**Figure 8:**
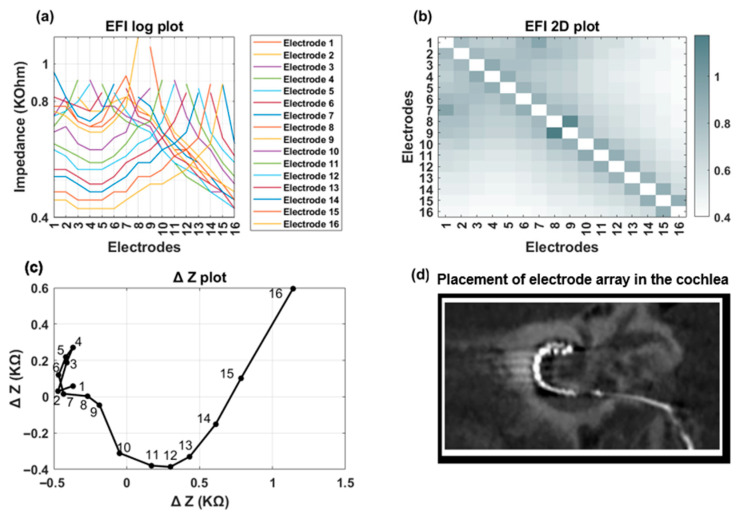
Clinical case with confirmed TFO on electrode 4. Measurements were taken three months after the surgery. (**a**) The line plot reveals a distinct on either side of the fold-over location (electrode 4); (**b**) The heat map highlights crosstalk between apical and less-apical electrode contacts; (**c**) The ∆Z plot demonstrates a sharp bend on electrode 4; (**d**) The CT image confirms the precise location of the TFO on electrode 4.

Figure 7 and Figure 8 showcase a clinical case featuring a TFO anomaly. EFI measurements and imaging were conducted at two distinct time points: immediately postoperatively and three months post-surgery. The line (Figure 7a and Figure 8a) and heat map (Figure 7b and Figure 8b) plots for both EFI measurements show an initial decrease in cross-impedance values as the distance from the stimulating electrode increases, followed by a subsequent rise. The ΔZ plot exhibits a distinct inflection at electrode contact 4 (Figure 7c and Figure 8c). Figure 7d and Figure 8d are CT images confirming the presence of a tip fold-over at electrode contact 4, corroborating the observations from the EFI-based TFO algorithm.

#### 3.2.2. EFI Without Imaging

A total of 118 cases out of 14,734, accounting for 0.80% of the analyzed data, were identified as having a TFO. Table 3 provides a detailed summary of the TFO occurrence rate, categorized by electrode array type. These data indicate that the incidence of TFO in pre-curved electrodes (96 out of 7842, 1.22%) is higher than that observed in straight lateral wall electrodes (22 out of 6892, 0.32%). Using a two-proportion Z-test, the calculated Z-score was approximately 6.15, corresponding to a *p*-value of <0.001. This indicates a statistically significant difference between the electrode array types with a TFO being more likely to occur in the pre-formed electrode array designs.

## 4. Discussion

Electric field imaging (EFI), also referred to as Trans Impedance Matrix (TIM) or Impedance Field Telemetry (IFT) by various cochlear implant manufacturers, has emerged as a promising tool for evaluating electrode placement. EFI measurement can be obtained during in-person or remote clinical appointments. The measurement takes only a few seconds to make, using regular clinical impedance measurement tools such as the AIM™ system or Target CI 1.5 onwards. Using the EFI matrix as an input, we developed an algorithm and implemented it in Target CI 1.6 to detect the presence of a TFO and also identify electrode contact location of the TFO. To evaluate the effectiveness of our TFO detection algorithm, we tested its performance across distinct scenarios: (1) plastic cochlea (2) temporal bone, and (3) patients with and without imaging confirmation of a TFO. This multi-faceted evaluation provides valuable insights into the algorithm’s accuracy and utility in both clinical and non-clinical settings. In our analysis of controlled samples and patient EFI data with imaging, the TFO detection algorithm demonstrated 100% accuracy in distinguishing between TFO and non-TFO cases. This performance is equivalent to, or better than, what has been reported in the previous literature for intraoperative data. When validated against intraoperative imaging, Cochlear Nucleus^®^ SmartNav System demonstrated 100% sensitivity and specificity in a single-center study of 113 ears [19] and 100% sensitivity with 99.6% specificity in a multicenter study of 484 insertions [20]. Earlier studies using the same TIM methodology include Kay-Rivest et al. [13], who reported 100% sensitivity and specificity in a smaller series, and Hoppe et al. [9], who reported a specificity of approximately 98.6% in a low-event-rate cohort.

It is noteworthy to mention that, in our study, EFI data with available imaging were collected as part of standard clinical care and not specifically intended to monitor TFO rate in Mid-Scala or SlimJ electrode array types. The instances where a TFO occurred was shared by the clinic to help evaluate the algorithm for detecting TFO other than identifying the TFO rate in Mid-Scala electrode arrays. One limitation is that the current intra-op EFI data with imaging may not provide precise information on the rate of TFO in these electrode types. Our findings in the much larger Target CI DataLake database (unconfirmed cases) align with previous studies, indicating a TFO occurrence rate of 0.80%. The main limitation of this large dataset is that it does not have imaging data to confirm either TFO or no TFO. This dataset also does not have fitting history changes. Some of these factors may influence the EFI measurement and algorithm performance.

Surgeons often choose between pre-curved and straight electrode arrays based on patient-specific factors and surgical objectives. Pre-curved electrode arrays are designed to position the electrodes closer to the modiolus. In contrast, straight electrode arrays are designed for lateral wall placement. When examining the influence of electrode array type, a significant difference in TFO prevalence emerges. Pre-curved electrode arrays exhibit a notably higher incidence of TFO compared to straight lateral wall electrode arrays, consistent with prior research. In this study, there is a significant difference between electrode array types, with pre-curved electrode arrays having a higher (1.22%) TFO prevalence than straight lateral wall electrode arrays (0.32%).

## 5. Conclusions

This study demonstrates that the Target CI 1.6 TFO algorithm, based on clinical standard of care impedance recordings, offers an effective and reliable method for detecting electrode array tip fold-overs in cochlear implants. Integrating such algorithms into intraoperative and fitting software platforms could enable rapid and accurate screening for TFO, alerting surgeons of cases where imaging or electrode repositioning might be necessary in the OR. This advancement has the potential to reduce the incidence of revision surgeries, lower healthcare costs, and significantly minimize patient discomfort.

## Figures and Tables

**Table 1:** Confusion matrix for temporal bone experiment.

True Positives	False Positives	True Negatives	False Negatives	Sensitivity	Specificity
4	0	4	0	100%	100%

**Table 2:** Confusion matrix of patient with imaging confirmation.

True Positives	False Positives	True Negatives	False Negatives	Sensitivity	Specificity
4	0	222	0	100%	100%

**Table 3:** Percentage of TFO across Advanced Bionics electrode array types.

Electrode Array Type	Implant Type	Total No. Per Implant Type	Total No. Per Electrode Array Group	No. of Detected TFO Anomalies	Percentage
Pre-curved	HR90K Advantage	2606	7842	96	1.22%
	Ultra	554			
	Ultra 3D	4682			
Straight lateral wall	HR90K Advantage	595	6892	22	0.32%
	Ultra	412			
	Ultra 3D	5885			
**Total**			**14,734**	**118**	**0.80%**

## Data Availability

The patient data underlying this study cannot be shared due to ethical, legal, and privacy restrictions. The analyzed data is available in tables of the paper.
